# Data and Tools Integration in the Canadian Open Neuroscience Platform

**DOI:** 10.1038/s41597-023-01946-1

**Published:** 2023-04-06

**Authors:** Jean-Baptiste Poline, Samir Das, Tristan Glatard, Cécile Madjar, Erin W. Dickie, Xavier Lecours, Thomas Beaudry, Natacha Beck, Brendan Behan, Shawn T. Brown, David Bujold, Michael Beauvais, Bryan Caron, Candice Czech, Moyez Dharsee, Mathieu Dugré, Ken Evans, Tom Gee, Giulia Ippoliti, Gregory Kiar, Bartha Maria Knoppers, Tristan Kuehn, Diana Le, Derek Lo, Mandana Mazaheri, Dave MacFarlane, Naser Muja, Emmet A. O’Brien, Liam O’Callaghan, Santiago Paiva, Patrick Park, Darcy Quesnel, Henri Rabelais, Pierre Rioux, Mélanie Legault, Jennifer Tremblay-Mercier, David Rotenberg, Jessica Stone, Ted Strauss, Ksenia Zaytseva, Joey Zhou, Simon Duchesne, Ali R. Khan, Sean Hill, Alan C. Evans

**Affiliations:** 1 McGill University, Montreal Neurological Institute and Hospital, McConnell Brain Imaging Centre, Neuro Data Science ORIGAMI lab, Montreal, Quebec Canada; 2 McGill University, Healthy Brains Healthy Lives, Neurohub, Montreal, Quebec Canada; 3 McGill University, McConnell Brain Imaging Centre, Montreal, Quebec Canada; 4 McGill University, Ludmer Centre for Mental Health, Montreal Neurological Institute, McGill Centre for Integrative Neuroscience, Montreal, Quebec Canada; 5 Computer Science, Concordia University, Montreal, Quebec Canada; 6 Krembil Centre for Neuroinformatics, Toronto, Ontario, Canada; 7 Ontario Brain Institute, Toronto, Ontario, Canada; 8 Hewlett Packard Entreprise, Pittsburgh, Pennsylvania US; 9 McGill University, Canadian Centre for Computational Genomics, Montreal, Quebec Canada; 10 University of Toronto, Toronto, Ontario, Canada; 11 Indoc Research, Toronto, Ontario, Canada; 12 McGill University, Department of Bioengineering, Montreal, Quebec Canada; 13 McGill University, Centre of Genomics and Policy, Montreal, Quebec Canada; 14 University of Western Ontario, Robarts Research Institute, Montreal, Quebec Canada; 15 McGill University, Healthy Brains Healthy Lives, Montreal, Quebec Canada; 16 Douglas Mental Health University Institute - Research Centre, StoP-Alzheimer Centre, Montreal, Quebec Canada

**Keywords:** Neuroscience, Research data

## Abstract

We present the Canadian Open Neuroscience Platform (CONP) portal to answer the research community’s need for flexible data sharing resources and provide advanced tools for search and processing infrastructure capacity. This portal differs from previous data sharing projects as it integrates datasets originating from a number of already existing platforms or databases through DataLad, a file level data integrity and access layer. The portal is also an entry point for searching and accessing a large number of standardized and containerized software and links to a computing infrastructure. It leverages community standards to help document and facilitate reuse of both datasets and tools, and already shows a growing community adoption giving access to more than 60 neuroscience datasets and over 70 tools. The CONP portal demonstrates the feasibility and offers a model of a distributed data and tool management system across 17 institutions throughout Canada.

## Abstract (summary)

**Table:** 

Measurement(s)	Digital Data Repository
Technology Type(s)	Digital Data Repository

## Introduction

Funding agencies, institutions and publishers are increasing pressure on the research community to make data findable, accessible, interoperable and reusable^1^, pushing beyond the individual researchers’ will to share their data^2^, in an effort to make research more reproducible and more efficient. This, in turn, has fostered the development of many data repositories in which researchers can host their datasets. For instance, in the field of neuroscience, the National Institutes of Health (NIH) recently published a request for applications to develop “web-accessible data archives to capture, store, and curate data related to BRAIN Initiative activities”^3^. The increasing number of data sharing platforms consequently makes data access standardization more and more desirable.

Issues in reproducibility, that have become apparent in recent years, have propelled the creation of numerous sharing infrastructures, including general-purpose examples such as Zenodo^4^ (https://zenodo.org/) or the Open Science Framework^5^, as well as specialized instances such as OpenNeuro^6^, LORIS^7^, BrainCode (https://www.braincode.ca) or XNAT^8^. Platforms often fall into one of two categories: large infrastructures with little constraints on the type of shared data or required metadata, or well-curated, more specialized repositories. Each has its strengths and weaknesses, and its utility depends on the scope of the projects.

Despite this progress in data findability and accessibility, it remains difficult for researchers to discover and reuse specialized datasets in neuroscience (e.g., “findability” dimension), particularly where access is controlled and constrained by specific ethical and legal considerations. Data sharing infrastructures vary widely in nature between scientific communities, and few are targeting the needs of the neuroscience community both in terms of “capacity to share” and “capacity to reuse”, which require access to both metadata and raw data. The prevalent model is one where sharing platforms require data to be moved to a central location from which access is provided, entailing duplication, versioning, and governance issues at the user’s end. In addition, most infrastructures are not specifically connecting data with the computing resources to usefully exploit these data (see the BrainLife project for a notable counterexample^9^).

The Canadian Open Neuroscience Platform (CONP – http://conp.ca) has been created to address these issues by facilitating open or restricted data and tool sharing among researchers in a well-grounded ethical and governance framework. The project is organized around four committees (Technical, Ethics and Governance, Training, and Communication) overseen by a Steering Committee. The CONP web portal (http://portal.conp.ca) integrates several open-source technologies to provide: i) extensible distributed federation of datasets, ii) unified search capabilities for data and software tools, and iii) the ability to run analyses either on High-Performance Computing (HPC) infrastructures or locally. The portal includes substantial training material developed by the CONP Training Committee, and its terms of use take into consideration ethical, legal, and governance constraints regarding data reuse identified by the CONP Ethics and Governance Committee.

The CONP targets both neuroscience and clinical researchers across the research communities, beginning with the Canadian research community. It offers a platform for both large datasets from “big data” laboratories and more clinical (smaller sample) datasets from other laboratories. With both a graphical user interface (GUI) and a command-line interface (CLI), the platform addresses varied needs within the neuroscience research community. Whether someone is searching for data, querying metadata, sharing results, or even processing datasets, CONP is designed with these capabilities in mind.

This paper outlines the portal’s design choices and highlights the current impact of the CONP portal on data and tool sharing in the neuroscience community. To facilitate reuse and interoperability, the CONP portal adopts the FAIR principles (Findable, Accessible, Interoperable and Reusable^1^), complies with best practices in data sharing^10^, and adopts existing dataset and software tool descriptor formats: the Data Tags Suite model (DATS)^11^ for data provenance and description, and Boutiques^12^ for containerized software tools. Given that it addresses a broad neuroscience community, the CONP does not enforce file formats other than for metadata descriptors, and it provides both a command-line and a web interface. The design of the portal considered usability and reusability, technology robustness, resources and time constraints, and interoperability and integration with existing software tools and environments through the use of current standards and software components.

## Methods

The platform design relies on open formats, APIs, and standards to allow for extensibility and promote interoperability. The key design ideas are based on the following constraints and considerations:

- The platform should integrate data resources from different infrastructures,
- Data and tools should be integrated without undue duplication,
- Integration of data and tools in the platform by community members should be feasible,
- Datasets and processing tools should implement the FAIR principles,
- Data governance should remain with the original data stewards or providers,
- The platform should rely on open formats and standards to foster reuse and integration with other projects, and
- The portal should provide intuitive navigation and provide users with documentation and help resources.

**Fig. 1:**
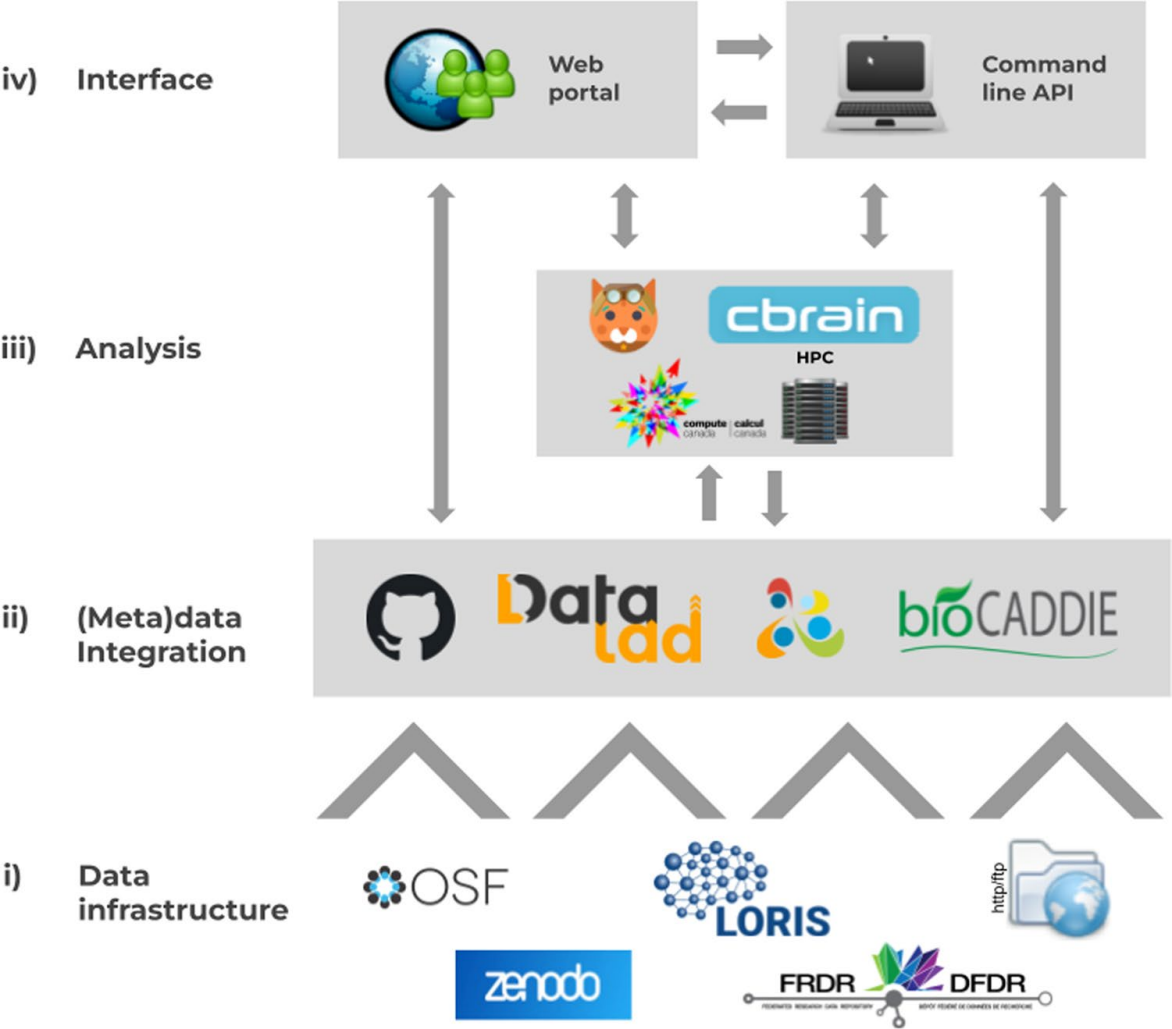
Architecture of the Canadian Open Neuroscience Platform. The platform is comprised of multiple tiers including: (i) Independent data infrastructure; (ii) Metadata integration across tools and datasets via standard models (Biocaddie DATS, Boutiques descriptors); (iii) Data analysis on High-Performance Computing and; (iv) Web and command-line interfaces.

The CONP consists of several key components (Fig. 1, see also Table 4):

I. A data infrastructure layer, incorporating disparate independent data repositories (e.g., Zenodo, www.zenodo.org, LORIS^7^, or the Open-Science Framework – OSF^5^);
II. A (meta)data integration layer, leveraging DataLad^13^, GitHub^14^, Boutiques tool descriptors^12^, enabling uniform data search queries based on the Data Tags Suite (DATS) model^15^;
III. An analysis layer that allows for simple download of tools and easy use of High-Performance Computing (HPC) environments; and
IV. An interface layer, which controls the interaction between these components and will be outlined further in the Results section.

### Data infrastructure

The CONP takes advantage of distributed data repositories, each with their own infrastructures, access control requirements, APIs, and licensing. This importantly gives flexibility to manage collections using specific context-appropriate tools, rather than prescribing an incomplete one-size-fits-all solution. The CONP presently supports accessing and integrating data from several flexible domain-agnostic datastores (OSF, Zenodo, FRDR-DFDR, https://www.frdr-dfdr.ca/), specific brain imaging repositories (LORIS, XNAT, Brain-CODE), and the commonly used HTTP and FTP web protocols. This set of supported infrastructures is intentionally extensible to any other repository which allows access via programmatic web-compatible interfaces (e.g. a RESTful API).

### Data integration

To integrate datasets across infrastructures, the CONP uses DataLad as a backend and GitHub to host the metadata. Crawlers automate both the discovery of tools (on Zenodo, www.zenodo.org) and datasets (on Zenodo and OSF) and the DataLad and GitHub integration workflows. CircleCI^16^ continuously tests if datasets are available and if data are accessible by testing the download of a few files from the datasets.

#### Integration of distributed datasets

The CONP adopts a decentralized architecture, to accommodate the various governance, ethical, and performance models required by data owners. For instance, some datasets may not easily be stored outside of the jurisdiction where they were acquired, while some institutions require local control of data storage, with some projects preferring to remain in control of access rules. This is all possible in CONP, as data can remain hosted anywhere on the internet.

Integration between datasets is provided by DataLad, a software library for managing Git repositories that references data. In DataLad, datasets are described in a Git repository containing metadata, file URLs and hashes of data blobs managed by git-annex. Importantly, a DataLad dataset does not generally contain the data themselves, which remain stored remotely. DataLad datasets can also be nested to represent dataset aggregation.

The CONP dataset consists of a main DataLad dataset and its metadata stored on GitHub (github.com/CONP-PCNO/conp-dataset) and referenced in the main DataLad index (http://datasets.datalad.org). The use of GitHub enables a variety of features useful for open-source software development; including issue tracking, code reviews, pull requests, branch protection, and integration with various applications. Datasets are integrated as Git submodules of the main dataset, and may be hosted on GitHub or on any other platform including GitLab or even a simple web server. This has the added benefit of being able to point to a specific commit, allowing continued evolution of the remote subdataset while the CONP portal keeps a reference to the stable version of the root dataset. Any DataLad dataset can be integrated into CONP provided that it contains a README file and a Data Tags Suite (DATS^17^) model file describing it. In addition, a configuration script can be added to the root of the dataset, to perform any required initialization.

The data themselves can be stored in any server implementing a protocol supported by git-annex, including HTTP, FTP, and many more. We used this flexibility to integrate data coming from three main types of sources. First, brain data archives such as the LORIS^7^, XNAT^18^, and Brain-CODE^19^ platforms provide a complete neuroscience data management solution for data ingestion, quality control, visualization, access control, and querying. They are commonly used to support large-scale multi-site longitudinal studies with hundreds of participants. Second, multi-disciplinary research data archives such as Zenodo in Europe, the Open Science Framework in the USA^5^, and the Federated Research Data Repository (FRDR)^20^ in Canada, provide simple ways to share research data publicly through the web and to guarantee long-term archival, findability, and immutability of data objects through Digital Object Identifiers (DOIs). They are typically used for local studies or companion data to a publication. Third, simple internet hosts accessible through the HTTP or FTP protocol allow for flexible integration of any other data already available online. CONP also provides local data-hosting for users who do not have the resources to make use of these other options.

Through git-annex, DataLad also supports authentication protocols, a critical feature for the ethical sharing of neuroscience data with restricted access. We extended this capability to support authentication to the LORIS, Zenodo, and OSF platforms. LORIS uses a common username/password authentication, which could be added to DataLad without particular challenges. Zenodo, however, implements private data sharing through secret tokens added to the file URLs. Since the file URLs are part of the DataLad repository and are therefore publicly shared, we implemented a custom mechanism to add and remove tokens from URLs on demand.

#### Data crawlers

To leverage the capabilities of existing research data archives (currently Zenodo and OSF, and in the future FRDR), we developed a crawling framework to manage the life cycle of DataLad datasets on GitHub. As a result, users can upload datasets to the CONP through these web platforms, without having to install and learn DataLad, or to become familiar with our GitHub workflow. The CONP data crawler performs the following actions, implemented as a base class containing most of the GitHub and DataLad logic, and as a set of derived classes containing the API calls specific to each crawled platform:

1. Search for CONP-tagged datasets in web platforms;
2. When a new dataset is found, create a new DataLad dataset;
3. When a dataset modification is detected, update the corresponding DataLad dataset;
4. Push modifications to CONP forked GitHub repository;
5. Create a pull request for each modified dataset, for the CONP maintainers to review and approve.

In addition, if no DATS model is found in the datasets, one is created automatically from the fields available in the web platforms, with minimal information such as title, license and creators.

#### Dataset testing suite

The CONP includes a dataset testing suite to mitigate the reliability challenges of decentralized systems. We implemented the testing suite in the CircleCI platform, due to its support for multithreaded testing, FTP connections, and interactive SSH sessions in testing environments. Hosting CONP DataLad datasets on GitHub allows for transparent integration with CircleCI. Similar to a software repository, dataset tests are triggered with every GitHub pull request, and their successful execution is required for the pull request to be approved by the maintainers. To reduce execution time, the testing framework only runs the tests for the datasets influenced by the pull request.

Datasets may become unavailable for a variety of transient reasons, including network interruptions, operational downtimes of the hosting platforms, or configuration errors. To detect these issues, we configured CircleCI to periodically test all the datasets available through the CONP every four hours, providing continuous monitoring. Results of this periodical testing are stored in CircleCI artifacts and are automatically displayed as status badges in the CONP portal. To increase robustness against transient errors, we used Pytest’s flaky module to re-run tests three times upon failure, with a 5-second delay.

The test suite tests the following properties for every dataset:

- Presence of a README file at the root of the dataset,
- Presence of a DATS model complying with our extended schema,
- Successful installation of the dataset with DataLad,
- Integrity of the git-annex repository, and
- Successful download of the four smallest files from a sample to reduce runtime.

For datasets that require authentication, we include credentials through CircleCI environment variables. To reduce the associated security risks, we configured the testing framework to skip the testing of authenticated datasets in pull requests. From these environment variables, the testing framework generates a DataLad authentication provider (LORIS, Brain-CODE), or configures the dataset to use access credentials (Zenodo). The testing suite is executed in a Docker container also available for download to replicate the testing environment.

#### Metadata integration

As the CONP portal brings together two types of research objects, software tools and datasets, we have reused two metadata standards developed to document these objects. The Boutiques standard^12^ describes a tool’s execution, inputs and outputs. We chose the Data Tags Suite (DATS) model^17^ developed by the BioCaddie consortium (Big Data to Knowledge NIH funds) to build the Datamed (https://datamed.org/) platform, for dataset description. Datamed was designed to be an equivalent of PubMed for datasets^21^, and DATS follows the architecture of the Journal Article Tag Suite – JATS. This choice was driven by the flexible nature of DATS and its associated material (e.g., validator, documentation). DATS also has the capacity to represent sub-datasets, a feature that can be used in association with the DataLad sub-datasets mechanism (implemented with git submodules).

These two standards are used to extract information about the research objects to be displayed in the portal, as well as provide the necessary JSON-LD information for making the datasets discoverable by Google Dataset Search. The DATS model also allows for an RDF representation of the information, which enables integration of the CONP metadata as a knowledge graph in BlueBrain Nexus^22^. The portal includes an advanced search interface mapped to a BlueBrain Nexus SPARQL endpoint where the DATS model files are regularly exported.

The DATS model contains a number of required fields: the name and description of the dataset, the name and affiliation(s) of the individual(s) who generated the data, the license under which a dataset is released, keywords, and data types and formats. It may also include details regarding related publications, funding bodies, and cross-referencing derived datasets. We have also applied the extensibility of the DATS model to add specific fields such as a structured record of the dataset’s source, allowing searches by institution, city, or country of origin^17^.

### Analysis & Tools

The CONP portal goes beyond the findability of tools, directly integrating tools into workflows and enabling their execution on HPC systems.

#### Tools sharing

Analysis tools are uniformly described in Boutiques, an open specification and software library for sharing tools according to the FAIR principles^1^. Boutiques descriptors are JSON objects containing a specification of the tool input data, parameters, and output data. They link to a Docker or Singularity container image where the tool and all its dependencies are installed and configured for execution. Boutiques tools can be reused in various platforms, such as workflow engines, as exemplified in TIGR-PURR (https://github.com/TIGRLab/TIGR_PURR), or in web platforms such as CBRAIN^23^ or VIP (https://www.creatis.insa-lyon.fr/vip/).

Boutiques tools can be published, archived, and retrieved in the Zenodo research archive or in the OpenAIRE-Nexus project. Once published, Boutiques tools receive a DOI, which makes their archives permanently findable.

#### Pipeline execution

Similar to the data integration layer, tools can be executed through both command-line and web interfaces. The Boutiques command-line tool can be used to run the tools locally with a uniform interface, provided that a container engine is installed. This is useful for testing analyses or processing smaller datasets. CONP datasets can be downloaded locally for processing through the DataLad command-line or Python API. Boutiques’ Python API also enables tool integration in external pipeline engines such as Pydra^24^, Nextflow^25^, or Apache Spark^26^.

For use-cases that benefit from the use of HPC clusters, the Clowdr command-line tool and Python API^27^ can easily be used to apply Boutiques tools concurrently to multiple subjects on HPC clusters available through the SLURM workload manager, such as the ones provided by Compute Canada (https://www.computecanada.ca/), or on the Amazon Elastic Computing Cloud (EC2). This allows CONP users to leverage their own resource allocation and to process CONP datasets through the DataLad interface.

Many CONP tools are also installed in CBRAIN^23^, a web portal interfaced with storage and computing resources at HPC centers, to provide a higher-level interface for users who do not want to use the command-line, or for developers who prefer to interact with HPC resources through a web API. CBRAIN can import Boutiques descriptors, and create web forms and HPC jobs to launch and monitor the tools. Pipelines that were installed as Docker images are converted to Singularity for deployment on HPC clusters. To facilitate the processing of datasets accessible via the CONP, CBRAIN also interfaces with DataLad, downloading files on-demand for processing.

Running a data analysis pipeline on CBRAIN requires a CBRAIN account. There is no current billing model associated with the compute part of the platform, because CBRAIN relies on academic computing resources, primarily Compute Canada, obtained through resource allocation competitions. The CBRAIN infrastructure allows for external compute resources to be attached to the platform such that an international research laboratory could use its own resources. CBRAIN also has a certain amount of computing time allocated on Compute Canada and when possible the team can offer these for reasonable usage. This needs to be directly requested to the CBRAIN infrastructure governance team through an email to CBRAIN support. Data derived from processing will be stored on the CBRAIN infrastructure, and the agreement by the CBRAIN team to process data will depend on both the compute time (if on CBRAIN Compute Canada allocation) and on the capacity to store these derived data. We note that CBRAIN can also attach data providers with the Principal Investigator’s own disk space allocation on Compute Canada, on other accessible infrastructures, or even on their own laboratory servers. Given the variety of situations, requests for compute time and disk space are handled on a case by case basis.

Finally, Boutiques executions, including local, Clowdr and CBRAIN ones, also collect anonymized provenance records for activity monitoring, traceability, and other applications.

## Results

The various technologies, methods, and design choices are integrated into a unified result, best described as the web layer of the CONP portal, available at http://portal.conp.ca. More general information about the CONP scope and goals is available at http://conp.ca. The current iteration of the portal, first launched in May 2020, is a platform that enables the sharing of structured data and tools. This includes data searches, uploading capabilities, analytics, filtering, querying, project, and provenance capabilities. The web layer is implemented with the Python Flask (https://flask.palletsprojects.com/en/1.1.x) framework and is designed to be used with any browser and operating system. The goal of the portal is to provide open and seamless access to researchers to search and download data, upload and share their own datasets and tools, as well as launch tools using numerous capabilities without requiring advanced computing skills.

### The CONP portal

Currently, the CONP portal has registered 73 datasets and 94 tools. Table 1 outlines summary statistics about the integrated pipelines and Table 2 reports on integrated tools.

**Table 1:** Summary statistics of integrated datasets (Jan 2023).

Data types	Datasets	Datasets requiring authentication	Files	Data size (GB)	Number of subjects or samples
Animal	12	1	29401	247.4	1780
Brain Disease	10	3	163,715	1,012	1,864
Brain Imaging	48	15	3,184,676	4500	8920
Cognition	4	2	85,049	583	1,325
Connectomes	7	0	22,386	1072	257
Electrophysiology	2	2	4,548	218	283
Genomics	6	0	57	27	2,508
Histology	14	0	35,243	1369.3	51
Quality Assurance	4	2	2,690,006	420	16
Transcriptomics	2	0	30	9	84

Out of the 73 datasets present in the CONP portal, 5 datasets are hosted in LORIS, 10 in BrainCode, 7 on OSF and 7 on Zenodo.

**Table 2:** summary of integrated pipelines by tags present in their Boutiques descriptor (Jan 2023).

Boutiques tags	Pipelines	Docker Images	Singularity images	Available on CBRAIN
neuroinformatics	50	39	11	9
mri	24	18	6	5
fmri	18	10	8	2
dmri, diffusion, diffusion MRI, dwi	12	12	—	1
other tags	47	46	1	—
bioinformatics	13	—	13	3
neuroimaging	48	41	7	1
blast	5	—	5	—
eeg	4	1	3	1

Before entering the portal, users must agree to the website’s Terms of Use, created by the CONP Ethics and Governance Committee to ensure that the data and resources will only be used for bona fide research purposes and that the confidentiality of participants whose data are on the portal is respected. For any given dataset, additional requirements and conditions can be attached through a specific data usage agreement document attached to that resource.

The portal is divided into 5 sections:

1. A Dashboard that contains key analytics summarizing the contents of the portal, as well as Spotlights and introductory information,
2. A Data section that contains a filterable listing of all datasets, including clickable structured descriptions of each dataset with more detailed provenance and download instructions,
3. A Tools and Pipelines section that similarly contains a searchable list of available resources, including detailed descriptors and options for launching,
4. A Share section that facilitates the upload of data, that interoperates with other platforms such as Zenodo and OSF; a web graphical interface to build the DATS model is also available in this section, and
5. An FAQ section for user support in addition to the Contact Us form.

Navigation of the portal was designed to be intuitive, but also fully documented in a way that is specifically designed and vetted for public consumption. With software, documentation is often notably lacking or insufficient, thereby rendering the tool/platform difficult or prone to error^28^. To address this issue, CONP was designed with structured documentation infused throughout the site. In addition, the CONP project has an important training and education component, and the CONP website gives access to content-rich tutorials, videos, and communication and feedback mechanisms. An indication of success would be to build a user community that integrates workflows with CONP, and would be easily quantifiable by the number of communication threads and channels revolving around the portal.

### Integrated datasets

A key dataset already exposed within this portal is PREVENT-AD (**P**re-symptomatic **E**valuation of **E**xperimental or **N**ovel **T**reatments for **A**lzheimer **D**isease, submitted), a cohort of cognitively healthy participants over 55 years old, at risk of developing Alzheimer Disease (AD) because their parents and/or siblings were/are affected by the disease. These ‘at-risk’ participants have been followed for a naturalistic study of the presymptomatic phase of AD since 2011 using multimodal measurements of various disease indicators. This is an example of interoperability principles outlined in Section 2.1., bridging CONP to existing database resources using web crawlers. The first open release of data was in April 2019 and comprises subject demographic information alongside comprehensive imaging data on 232 subjects. That dataset was updated in August 2020 with an extra 86 subjects for a total of 308 participants. Another release of data acquired on this cohort with more sensitive clinical patient information has been released under restricted access controls in November 2020. The registered release of PREVENT-AD includes cognition, cerebrospinal fluid protein levels, neurosensory measures, genetics and other clinical data. The imaging datasets of both releases have all been organized according to the BIDS standard.

Other notable datasets include template data, such as the “Multicenter Single Subject Human MRI Phantom” with several hundreds of scans longitudinally collected over 15 years, as well as the SIMON (“Single Individual volunteer for Multiple Observations across Networks”) dataset of more than one hundred scans of a single individual over 35 sites^29^. Additionally, CONP offers connections to open data releases on the Brain-CODE platform, including MRI imaging from over 30 mouse models related to autism^30^. Other releases currently available through Brain-CODE also include quality assurance data from several MRI scanners within Canada, and responses to a neurodevelopmental disorders priority setting partnership (https://braininstitute.ca/img/JLA-NDD-Final-Report.pdf).

While the focus of CONP is open data, we acknowledge that not every piece of associated data can be released openly. As such, we allow for the exposure of some data that requires authentication and we report the authentication model of datasets in their DATS descriptor.

### Integrated software tools

Numerous tools have already been incorporated within the CONP, including tools for functional and structural image processing, DWI, EEG, bioinformatics, as well as BIDS apps^31^. The portal initially serves as a registry for any tools available in the portal, where filters and searches can be used to locate a desired resource. However, capabilities exist to run these tools and pipelines directly on a local system or using cloud-based approaches. Table 2 summarizes the pipelines available on the CONP portal by Boutiques tag.

## Discussion

There is currently an increased awareness of the importance of data sharing within the neuroscience community (see for instance^3^) with specific focus in several areas, such as ethics and privacy, technological design, optimization of workflows, provenance capture and standardization, and security, including calls from funding agencies to promote data re-use and sharing. A platform can be designed with a number of use cases and adoption by different communities in mind. The CONP portal infrastructure holds a special place within the numerous data sharing initiatives specialized for neuroscience, neuroimaging or general purpose. To illustrate this, Table 3 compares the CONP portal to a sample of other data sharing platforms. Surveying the criteria outlined in the table, four main points summarize the CONP portal’s place in the landscape of data sharing platforms.

**Table 3:** Data sharing platforms comparison.

Platform	Storage Model	Research Focus	Access Control	Tool-Data Integration
CONP Portal	Decentralized	Neuroscience	Determined by storage provider	Boutiques
Zenodo	Centralized	General	Public or Restricted	None
OSF	Centralized	General	Public or Private	None
NeuroMorpho	Centralized	Digital neurons	Public	None
OpenNeuro	Centralized	BIDS Datasets	Public after embargo	BIDS Apps
NIMH Data Archive	Centralized	Human subjects	Restricted	None
FRDR	Centralized	General (Canada only)	Public after embargo	None
Harvard Dataverse	Centralized	General	Public with Restricted subsets	None

**Table 4:** technical glossary.

Component	Description	How it’s used in CONP
Boutiques	A standard to describe a pipeline of tool	CONP tools are described with a Boutiques descriptor
BrainCODE	A platform to share data in Ontario	A CONP backend infrastructure
CBRAIN	A web platform to launch pipelines	The CONP portal links to pipelines installed on CBRAIN
CircleCI	A continuous integration system	Used to test datasets availability and compliance to CONP metadata schema
DataLad	A distributed data management system	The backend layer for integrating datasets in CONP
DATS	A standard for describing datasets	CONP datasets are described with DATS
FRDR	A sharing platform for Canadian research data	A CONP backend infrastructure (work in progress)
Git-Annex	A Git extension to manage large datasets	Used as a backend by DataLad
GitHub	A web platform for Git repositories management	Hosts the CONP DataLad datasets
LORIS	A database system for multimodal data	A CONP backend infrastructure
Nexus	A Linked Data platform to represent metadata	DATS information are integrated in Nexus for more powerful searches
OSF	A web platform for permanent research data hosting	A CONP backend infrastructure
XNAT	A database system for neuroimaging	A CONP backend infrastructure
Zenodo	A web platform for permanent research data hosting	A CONP backend infrastructure

First, unique among the surveyed platforms is the CONP portal’s decentralized data storage model, where data are kept in their original infrastructures, but where metadata are both centralized and decentralized, as DataLad datasets are Git repositories. The separation of raw data and metadata is one of the critical design choices of the platform, allowing updates of the dataset description without losing direct access and integrity check mechanisms on the raw data.

The pros and cons of a decentralized approach depend on the type of usage. For CONP, we implemented data sharing using a distributed architecture such that institutions could maintain their local infrastructure data governance. Concurrently, we designed a portal that gives direct access to the data. This is done by registering datasets with DataLad, which effectively stores the location of the raw data in a Git repository. This implies that download of data has to be performed with DataLad, therefore relying on a local DataLad installation. DataLad has truly excellent installation documentation across platforms, but nevertheless this is a constraint and installation issues can occur. A web downloader that removes the need for any local installation has been developed for public datasets which can now be downloaded through the portal frontend. The pros of a fully centralized architecture are ease of access management and capacity to standardize datasets. CONP has chosen a path that makes community contributions possible while centralizing metadata.

In general, whole infrastructures’ (such as OpenNeuro) content are not integrated in CONP: the integration is done at the dataset level. We show however that we can effectively search across datasets hosted by various infrastructures. As noted above, a CONP dataset can only be integrated if the hosting infrastructure has a DataLad (GitAnnex) backend, and complies with the DATS requirements and mandatory fields, in particular the license or Data Usage Agreement (DUA) has to be included. While the particular DUA of an individual platform can hamper dataset integration, the general principle is that the platform on which the dataset is hosted will handle the access authorization. Only public metadata (handled by Git and not GitAnnex) are necessarily shared. The policy for datasets integration in the platform is as follows. When the integration requires some technical resources, the Steering Committee decides on the relevance of the dataset for the neuroscience community. For “discoverable” datasets for which no resources are needed (e.g. located on Zenodo) datasets are only checked for technical conformance and topic by the technical steering committee.

Second, the CONP platform specializes in Neuroscience datasets and tools. This is to maximize the usability and findability of datasets for this specific community, but without enforcing one specific file format or data structure, like other neuroscience-specific repositories do (e.g. BIDS), as there is no such generic standard across neuroscience datasets, acquisition instruments, neuroscience (sub) fields, etc. We adopted the DATS metadata model because it was developed by the life sciences community and is easily adaptable to the neuroscience field. Unlike more general metadata standards such as Dublin Core or bioschema used by more general-purpose data sharing platforms, the DATS model’s specialised features allow us to collect useful neuroscience-specific metadata. Nevertheless, other choices could have been made, and as long as there is a possible mapping between descriptors the platform should be able to interoperate with others.

Third, there is a clear need for a data sharing model that does not make all data public. Clearly, the more identifiable the data, the more access should be restricted. Beyond demographic information (names, date of birth, social security numbers) data may be identifiable when linked to additional data points, allowing for individuals to be reidentified^32^. The likelihood of reidentification increases with the amount of data accessible for a specific individual. These privacy concerns can restrict the diffusion of many clinical datasets which have potential for progress in disease understanding or prevention. It is therefore critical that solutions are found to share data securely with researchers with the appropriate ethical credentials. Another less fundamental aspect, but one that is pervasive, is that dataset stewards often require acknowledgement and records of usage. The CONP portal’s decentralized storage model allows data stewards to implement any data access protocol that fits their needs, while still conforming to a uniform search and access interface.

Fourth, the platform connects tools and datasets and enables searching these objects through a common interface, while considering different metadata models for datasets and tools. In general, it is difficult to tightly integrate tools and data unless a precise standard format is imposed on the data and tools have been specifically developed to account for the standard. The example of the BIDS apps is interesting in this respect, relying on a common library to interrogate data under the BIDS format (PyBIDS^33^) and the corresponding “BIDS apps” that can assume the BIDS dataset format. This, however, does not solve the general problem of associating neuroscience data with processing tools beyond some brain imaging data. A less stringent requirement could be achieved in the future by registering the link between the datasets and their processing tools using DataLad and Boutiques unique identifiers.

Boutiques, the metadata standard chosen for tools, offers a practical and simple way to describe the top level execution of a tool, and as such has been adopted by CBRAIN. In the future, treating containerized tools as “executable data” could lead to a common descriptor across data and “executable data”. The selection of software tools integrated in the platform is motivated by user needs. Integrating a new tool requires that it is (1) containerized using Docker or Singularity, (2) described in the Boutiques format. Both processes are well documented and supported by the CONP technical team. In general, the CONP tool integration model perfectly fits with publicly-available tools. Restricted tools, such as those that require a license and/or are not publicly available, can be integrated on a case by case basis through additional configuration. Technically, some well-structured tools such as BIDS apps could be automatically integrated using mechanisms similar to the dataset crawlers. In practice, manual intervention is still required to check security requirements, adjust tool parameter types and dependencies, and validate functionality.

The CONP portal largely adopts the FAIR principles for data as well as for software tools, namely Findability (F1-F4), Accessibility (A1-A2), Interoperability (I1-I3), and Reusability (R1.1-3)^1^. More specifically: (F1) data (with restrictions) and tools are assigned a globally unique and persistent identifier – a DOI created by Zenodo or the OSF, (F2) data and tools are described with rich metadata – through the DATS and Boutiques formats, (F3) metadata on tools clearly includes the identifier of the tool they describe – as part of the Boutiques format, and (F4) metadata are registered in a searchable resource – the CONP portal and the Nexus repository. It should be noted that due to the decentralized nature of the platform, some datasets (not tools) may not be assigned a persistent identifier as required by (F1) and (F3), due to lack of support by the data hosting backend. The CONP technical team is actively working on addressing this limitation. Regarding Accessibility, CONP guarantees that (A1) all data, metadata and tools are retrievable through a standard communication protocol – http, ftp or Git, and (A2) metadata are accessible on GitHub (data) or Zenodo (tools) even if the data are no longer available. In terms of Interoperability, (I1) DATS and Boutiques use JSON, a formal, accessible, shared, and broadly applicable language for knowledge representation, data use formats commonly used in neuroscience (NiFTI and MINC), and tools use standard container image formats (Docker and Singularity), (I2) metadata use vocabularies that are documented and resolvable using globally unique and persistent identifiers (DATS^34^, Boutiques^35^), and (I3) DATS and Boutiques include qualified references to other metadata whenever relevant. Finally, (R1.1) data are released with a clear and accessible data usage license, (R1.2) data may be associated with detailed provenance, through DataLad. Regarding (R1.3), no particular general metadata community standard currently exists in neurosciences, which justifies our adoption and customization of DATS.

CONP adapts to the needs of neuroscientists interested in data sharing for community-driven research collaborations. While the portal was fully released in May 2020, development is still ongoing and a variety of new features are expected in the near future.

We will further help the community integrate and document new datasets, increasing the amount of data and the variety of platforms through which data can be integrated into the portal. This will follow the expansion of possible connections of DataLad and/or Git-Annex to other backends (e.g. Dataverse). The use cases of CONP may also prompt some enhancement of DataLad, thus building an open source data sharing ecosystem.

We will also work on creating new datasets from derived data from existing datasets in the portal. Following the so-called “Yoda principles” proposed by Hanke and Halchenko^13^, a derived dataset should point to two sub-datasets, one consisting of the containerized tool and its descriptor, the other being the input dataset. The combination of tools and datasets within the same platform also lays out interesting directions for future work. For instance, we envisage building a recommendation system that could help users associate tools and datasets, by searching on the datasets and tools descriptors for possible associations. Another option is to allow the records of these derived datasets to be searched and indicate to users which tools have been run on a specific dataset, or inversely, which datasets have been processed by a specific tool. The “datalad run” command and the Boutiques bash interface share some features that could form a powerful ecosystem for reproducible and documented tool execution.

The curation of metadata associated with datasets and tools will constantly evolve. We already provide a first graphical interface that will help researchers to document a dataset, ensuring that required fields of the DATS model are entered. Since DATS can be easily turned into linked data representation (JSON-LD/RDF), these can be absorbed by Nexus and provide a powerful search engine across datasets.

New data sharing platforms need to be part of the larger ecosystem and interoperate with existing platforms. By choosing DataLad as its backend, the CONP portal makes it possible to easily integrate any neuroscience DataLad dataset, including OpenNeuro, and does not impose a specified format but still enforces the minimal amount of documentation to improve findability and reusability.

Lastly, we believe that this platform is ideally situated to foster the posting and review of data and tools, elevating these objects to published and citable research, through the traditional peer review system. The CONP portal already links to the NeuroLibre^36^ infrastructure for reviewing technical components of Jupyter Notebooks, and could streamline publication to other innovative publishing platforms such as the Organization for Human Brain Mapping’s Aperture (www.humanbrainmapping.org) publishing platform project.

## Data Availability

Data are available through either a direct download or through the DataLad backend. Data usage agreement and accessibility depend on the dataset.

## Code availability

The code used for the portal is available on https://github.com/CONP-PCNO/conp-portal and a version of the code is available on Zenodo^37^.
